# Mental health research capacity building in sub-Saharan Africa: the African Mental Health Research Initiative

**DOI:** 10.1017/gmh.2019.32

**Published:** 2020-03-02

**Authors:** Dixon Chibanda, Melanie Abas, Rosemary Musesengwa, Chris Merritt, Katherine Sorsdahl, Walter Mangezi, Chiwoza Bandawe, Frances Cowan, Ricardo Araya, Exnevia Gomo, Lorna Gibson, Helen Weiss, Charlotte Hanlon, Crick Lund

**Affiliations:** 1 Department of Psychiatry, College of Health Sciences, University of Zimbabwe, Harare, Zimbabwe; 2 Institute of Psychiatry, Psychology and Neuroscience, King's College London, London, UK; 3 Department of Psychiatry and Mental Health, Faculty of Health Sciences, University of Cape Town, Observatory, South Africa; 4 College of Medicine, University of Malawi, Blantyre, Malawi; 5 Liverpool School of Tropical Medicine, Liverpool, UK; 6 Department of Immunology, College of Health Sciences, University of Zimbabwe, Harare, Zimbabwe; 7 Department of Epidemiology, London School of Hygiene and Tropical Medicine, London, UK; 8 MRC Tropical Epidemiology Group, London School of Hygiene and Tropical Medicine, London, UK; 9 Department of Psychiatry, College of Health Sciences, Addis Ababa University, Addis Ababa, Ethiopia; 10 Department of Mental Health, Faculty of Health Sciences, University of Cape Town, Observatory, South Africa

**Keywords:** Africa, AMARI, capacity building, mental, neurological, substance use disorders

## Abstract

Mental, neurological and substance use (MNS) disorders are a leading, but neglected, cause of morbidity and mortality in sub-Saharan Africa. The treatment gap for MNS is vast with only 10% of people with MNS disorders in low-income countries accessing evidence-based treatments. Reasons for this include low awareness of the burden of MNS disorders and limited evidence to support development, adaptation and implementation of effective and feasible treatments. The overall goal of the African Mental Health Research Initiative (AMARI) is to build an African-led network of MNS researchers in Ethiopia, Malawi, South Africa and Zimbabwe, who are equipped to lead high quality mental health research programs that meet the needs of their countries, and to establish a sustainable career pipeline for these researchers with an emphasis on integrating MNS research into existing programs such as HIV/AIDS. This paper describes the process leading to the development of AMARI's objectives through a theory of change workshop, successes and challenges that have been faced by the consortium in the last 4 years, and the future role that AMARI could play in further building MNS research capacity by brining on board more institutions from low- and middle-income countries with an emphasis on developing an evidence-based training curriculum and a research-driven care service.

## Background

Mental, neurological and substance use (MNS) disorders are a leading, but neglected, cause of morbidity and mortality in sub-Saharan Africa (SSA) (Charlson *et al*., 2014). MNS disorders account for >25% of all years lived with disability globally, more than cardiac disease or cancer (World Health Organization, 2010; Whiteford *et al*., 2016). The treatment gap is vast, only 10% of people with MNS disorders in low-income countries access evidence-based treatments compared to 33% in high-income countries (Hyman *et al*., 2006; Shidhaye *et al*., 2015). Reasons for this include low awareness of the burden of MNS disorders and limited evidence to support development, adaptation and implementation of effective and feasible treatments. While pockets of mental health research excellence exist in Africa, MNS research capacity is generally limited, particularly in mental health intervention, service and system research. Mental health research excellence is currently undermined by restricted opportunities for training and mentorship, unclear career pathways, lack of integration in general medical settings, limited multi-disciplinary collaboration and the lack of a critical mass of MNS researchers and leaders (Liu *et al*., 2016).

The African Mental Health Research Initiative (AMARI) is a mental health research capacity building initiative funded through the Developing Excellence in Leadership, Training and Science (DELTAS) program through the African Academy of Sciences (AAS) which is supported by the Wellcome Trust and the UK Department of International Development. The AMARI consortium established in 2015 comprises four African universities [University of Zimbabwe, University of Cape Town (UCT), Addis Ababa University and College of Medicine Malawi], supported by three UK universities (King's College London, London School of Hygiene & Tropical Medicine and Liverpool School of Tropical Medicine). AMARI was built on the foundation of prior capacity building programs such as the EMERALD program at the UCT. The overall goal of the AMARI is twofold. First, to build a critical mass of an African-led network of MNS researchers in Ethiopia, Malawi, South Africa and Zimbabwe, who are equipped to lead high quality mental health research programs that meet the needs of their countries. Second, to establish a sustainable career pipeline for these researchers with an emphasis on integrating MNS research into existing programs such as HIV/AIDS, NCDs, maternal and child health (Chibanda *et al*., 2014).

AMARI seeks to achieve the above overall goals through four specific objectives: (1) select and train 47 MNS research fellows from a range of disciplines at Masters, Ph.D. and post-doctoral levels in research excellence; (2) build leadership skills of fellows through a Career Development Series on Leadership, Management and Mentoring; (3) design and test an advocacy and systems change strategy for the four countries which can be adapted for other African countries, with an aim to build sustainable career pathways in MNS academia and (4) develop a web-based support platform for training, supervision and networking.

## AMARI theory of change

**Fig. 1.**
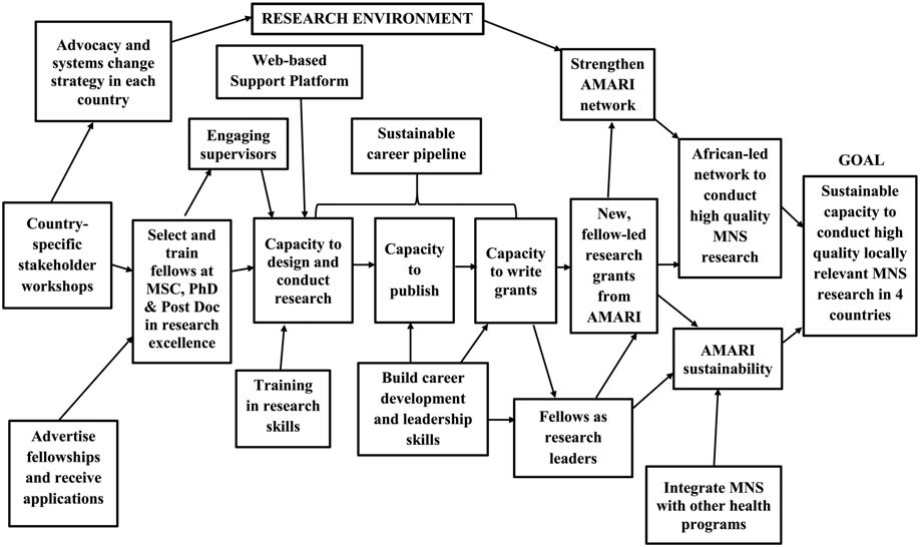
AMARI ToC.

A theory of change (ToC) workshop carried out during the first year of the program outlined the pathways to be followed to achieve these specific objectives. The ToC approach provides a framework for planning, implementation and evaluation of complex initiatives (Kubisch *et al*., 2001). In recent years, it has been used extensively in planning, implementing and evaluating mental health initiatives (Breuer *et al*., 2014; De Silva *et al*., 2014; Hailemariam *et al*., 2015; Chibanda *et al*., 2016*a*). The AMARI ToC was used to bring together key stakeholders in an effort to build consensus around a hypothesized causal pathway that would lead to the realization of AMARI's goals. The stakeholders were selected from all the four African countries in the consortium according to their involvement in Mental Health Research and their potential to keep AMARI sustainable through their institutions. A ToC map outlining key indicators, barriers, assumptions, rationale and other critical milestones was developed over a 5-day period by the AMARI collaborative group (Fig. 1). The AMARI ToC outlined strategies for addressing a wide range of issues related to the program objectives which included (i) developing the AMARI training courses, (ii) gathering baseline data for each country, for evaluation purposes, (iii) design of an advocacy and systems change strategy; (iv) conducting qualitative and quantitative interviews with local policymakers and service users to identify needs and priorities; (v) identifying and training supervisors and (vi) recruitment of AMARI fellows across the four countries.

Although the AMARI ToC can be viewed as the anchor for the program's direction it is a living document influenced by an iterative interaction between partners and key stakeholders with the final evaluation expected to be carried out in the next 2 years. We provide below some initial successes and challenges.

## AMARI successes and challenges

### Successes

As AMARI reaches its half-way mark, with 37 (two PostDoc, 25 Ph.D. and 10 MPhil) fellows recruited and registered across the four countries focusing on eight different research domains (Table 1), it is timely to reflect on some of the successes and challenges of the program so far. Key achievements have included (i) successfully stimulating interest in MNS research and recruiting 37 research fellows across the four countries as described in the ‘selection’ box in the ToC (Fig. 1), (ii) establishing formal networks within institutional partners with terms of reference, recruitment procedures and communications strategies ‘engagement’, (iii) recruitment of Fellows and understanding their background per cohort as a way of developing their specific career pipelines, (iv) putting in place supervisory arrangements with both local and external supervisors including frequency of meetings and reporting and (v) fellows applying and successfully securing additional grant funds for extra skills building, training, travel, operational costs and networking. These achievements are described briefly below. A detailed description is intended for publication upon completion of the program within the next 2 years.

**Table 1.** AMARI research domains

Area of research	Focus
Tool validation	Screening tools for MNS used by lay health workers
Maternal MH	Antenatal and postnatal depression in primary care
HIV and MH	Depression in PLWH attending primary care services
Neuro-cognitive assessment	HIV and Stroke associated neuro-cognitive impairment
Deliberate self-harm	Risk factors for deliberate self-harm among young people
Non-communicable diseases	Diabetes, hypertension and depression as co-morbidities
Psycho-pharmacology	Interaction of ARVs with psychiatric medication
Intervention development	Effective use of lay health workers for task shifting

### AMARI innovative courses

The *Academic Competencies Enhancement Series* (ACES) courses, which falls under sustainable career pipeline in the ToC (Fig. 1), focuses on building technical research and career building skills over 10 workshops (Table 2) which focus on three key themes: writing skills, self-development and science engagement. Writing skills focus on grant writing and academic writing for peer review journals. Self-development focuses on mentoring training, team working, work–life balance and career strategy. The engagement workshops focus on presentation skills, use of digital media, community and stakeholder teaching and engaging policymakers.

**Table 2.** Key components of the ACES program

Area covered by ACES	Duration of training	Online or face to face
Presentation skills	1 day	Face to face
Mentoring skills	1 day	Face to face
Research career strategy	1 day	Face to face
Using media to disseminate research	2 h	Online and face to face
Working in a team	¾ day	Face to face
Work-life balance	¾ day	Face to face
Introduction to teaching adults (in low-resource settings)	2 h	Online
Introduction to health systems and engaging policy makers	2 h	Online
Introduction to grant writing (early career focus)	2 h	Online

The *AMARI MPhil Webinars and Teaching Program* run from the University of Cape Town (UCT) has formed the basis of the consortium's monthly webinars which fall under capacity to design and conduct research in the ToC map (Fig. 1). Building on the existing infrastructure and content of the monthly webinars run through UCT, fellows have had access to the online interactive platform through the existing Public Mental health program running through the UCT. This MPhil program is currently the only one of its kind in Africa. Through AMARI funding access to this program has been made possible for fellows who would otherwise be unable to afford the fees and travel to the UCT. The UCT MPhil application is open to all applicants from AMARI country partners offering a Masters level training for mental health services research and an entry to further Ph.D. studies.

The *AMARI Public engagement grant – ARTICULATE* public engagement component, builds on the engagement courses provided through ACES. This is reflected under ‘fellows as leaders’ in the ToC (Fig. 1). ARTICULATE provides an interactive platform for AMARI fellows, the community and key stakeholders, aimed at strengthening existing, cultural and or arts initiatives that work with or are prepared to work with people living with MNS in the four countries. In all four countries AMARI has identified and engaged existing arts and cultural initiatives where fellows are formally attached and interact with user groups and community members to increase awareness around mental health issues. This process is being documented through a four-part documentary film and a 20-series radio program run over 3 years. The documentary and radio programs will run in all four AMARI African countries.

### Challenges

AMARI has in the last 3 years made considerable progress in recruiting and training Masters, Doctoral and Post-doctoral Fellows, whose research is contributing toward narrowing the treatment gap for MNS disorders in the four countries. A critical challenge has been the poor basic research skills in Ph.D. and MPhil candidates suggesting a need to focus on basic research training. Some challenges that have emerged include stimulating interest in MNS research for post-doc fellows, reflecting the underdevelopment of MNS research in two of the countries (Malawi and Zimbabwe) where ‘selection and training’ of post-doctoral fellows has not happened as described in the ToC (Fig. 1). The post-doctoral recruitment has, however, been successful where established research programs exist, for example, in Ethiopia and South Africa where both partners are WHO collaborating centers for mental health research. Another challenge has been identifying and engaging suitable local supervisors (Fig. 1) with relevant mental health and research experience. While there is supervisory support from the AMARI UK institutions managing the relationship between local and external (UK) supervisors has at times been difficult due to differing expectations and pre-existing experience as well as institutional structures which have not always been aligned with AMARI's objectives which encourage multidisciplinary Ph.D.s. The recruitment of fellows and registration at the institutional level has at times been delayed due to institutionally based administrative barriers such as lack of a post-doctoral career pathway and prolonged ethics approval. Operationalizing some of the AMARI developed online systems such as the Monitoring and Evaluation online log book, running the Webinar Series has been challenging due to unreliable and poor internet connectivity in some AMARI institutions. Finally, the proportion of clinicians applying for the program has been low with only six (16%) clinicians applying for either the MPhil, Ph.D. or post-doctoral positions. There is therefore a need to look into ways of making research a critical component of clinical work in SSA (Abas *et al*., 2014) by focusing on research that can be translated into clinical practice and contribute toward narrowing the treatment gap for MNS.

## Future of AMARI

During a recent mid-term evaluation held in South Africa for the AAS DELTAS programs, AMARI was found to be on track in relation to the key objectives described above. As the program begins to look into the future a number of issues have emerged through consultations with stakeholders. These include the opportunity to bring on board more African countries as the program becomes consolidated across SSA. Interest from institutions outside Africa from low- and middle-income countries (LMIC) such as India and China have opened up discussions about possibility of a broader global initiative.

**Fig. 2.**
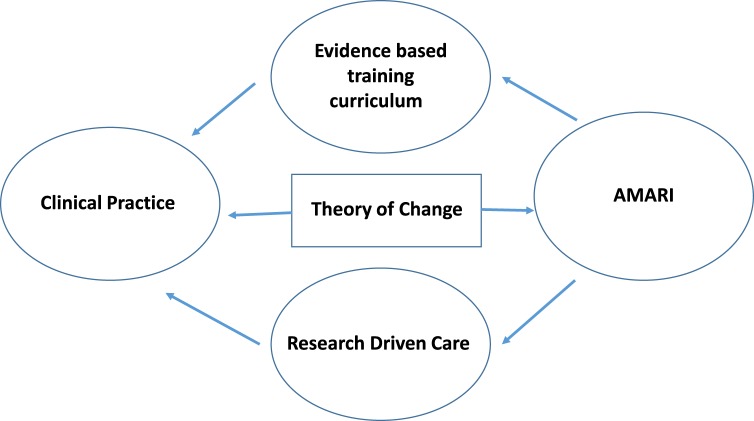
AMARI future role.

Common themes and challenges across LMIC such as the large treatment gap for MNS (Patel *et al*., 2010) put AMARI in a unique position to lead the expansion of this capacity building initiative with an emphasis on developing excellence in leadership, training and science in a sustainable way. AMARI has in the last 3 years focused on critical MNS research areas identified as priority areas in SSA (Hakim *et al*., 2017). Moving forward, AMARI will have to build on the eight research domains by using this new knowledge to drive development of training curriculum and research driven care to influence clinical practice that ultimately contributes toward the narrowing of the treatment gap for MNS disorders. In addition, AMARI will need to focus on biomedical research in line with current debate on etiology and classification of mental disorders as outlined by the research domain criteria (Cuthbert and Insel, 2013). Although clinicians have not been the largest beneficiaries of AMARI, in the future AMARI will have to engage clinicians through appropriate platforms such as ToC workshops, to be better informed of the challenges that clinicians may be facing. These challenges could then be addressed using empirical observation and appropriate research methods (Fig. 2). Furthermore, the introduction of the MD-Ph.D. courses which equip the clinician with research skills will be needed including early identification of promising medical students who could be part of clinical research teams. AMARI could initially facilitate ToC workshops to inform the program of the needs of various clinical personnel starting with medical students. This knowledge could then be synthesized to develop an evidence-based training curriculum and a research driven care platform which would seek to strengthen clinical practice (Fig. 2).

For instance, with the growing evidence of task-shifting approaches (Petersen *et al*., 2012; van Ginneken *et al*., 2013; Joshi *et al*., 2014; Nakimuli-Mpungu *et al*., 2015; Chibanda *et al*., 2016*b*; Javadi *et al*., 2017; Patel *et al*., 2017) AMARI could in the future contribute toward the development of culturally appropriate training curriculum and research strategies aimed at ensuring sustainable implementation and scale up of evidence based care as has been the case in the fight against HIV/AIDS (Zachariah *et al*., 2009; Swartz *et al*., 2014).

## Conclusion

Key innovative lessons from AMARI include (i) the focus on capacity building which is not only restricted to research methods, but also to leadership, career development and public engagement, (ii) the development of strong mutually beneficial partnerships between high and LMIC, (iii) the creation of opportunities for Fellows to become future leaders through a focus on key outcomes such as grants and publications and (iv) it is African led.
